# Phenotyping and Plant Breeding: Overcoming the Barriers

**DOI:** 10.3389/fpls.2019.01713

**Published:** 2020-01-09

**Authors:** Dionysia A. Fasoula, Ioannis M. Ioannides, Michalis Omirou

**Affiliations:** ^1^ Department of Plant Breeding, Agricultural Research Institute, Nicosia, Cyprus; ^2^ Department of Agrobiotechnology, Agricultural Research Institute, Nicosia, Cyprus

**Keywords:** honeycomb selection designs, interplant competition, prognostic breeding, genetic gain, prognostic equations, root phenotyping, crop wild relatives, epigenetics

## Introduction

Plant breeding is based on phenotyping, not only because of tradition but also because of essence. A plant phenotype is the result of the interactions between the genome of a stationary plant and all the micro- and mega-environments encountered during its life span. Over the recent years, we have witnessed an explosion in state-of-the-art technologies developed through collaborative efforts of multidisciplinary teams to assist the process of high-throughput plant phenotyping in plant breeding (NSF, 2011), first only under controlled conditions and, more recently, also under real field conditions (Lawrence-Dill et al., 2019).

Yet, plant phenotyping is still the bottleneck for breeding and farming (Chawade et al., 2019) and the average plant-breeding program has not been adopting the new developments adequately (Awada et al., 2018) Among the solutions proposed, a major international effort is being directed towards data and protocol standardization (Pieruschka and Schurr, 2019) that will be discussed later.

What so far has not been seriously considered, but we assert it should occupy a central part in the relevant discussions, is the choice of the appropriate unit of plant phenotyping in the field, so that the efficiency of selection in plant breeding programs and the corresponding measurable genetic gain are maximized. Should the community continue using the multi-plant, densely grown field plot as the unit of phenotyping and evaluation for plant breeding purposes or should we consider more efficient approaches based on the maximization of a plant’s phenotypic expression and differentiation?

To increase efficiency in plant breeding, we advocate that the most appropriate unit of plant phenotyping for selection purposes should correspond to the individual plant grown unhindered in the absence of competitive interactions so that phenotypic expression and the corresponding phenotypic variance are maximized, the coefficient of variation (CV) of single-plant yields is minimized, and spatial heterogeneity is effectively controlled. These conditions are met when plants are allocated in the field according to one of the honeycomb selection designs (HSD) (Fasoulas and Fasoula, 1995; Fasoula and Fasoula, 1997a; Fasoula and Fasoula, 2002). 

In this opinion paper, we present a list of some commonly encountered barriers during a plant breeding program, including the so-called pre-breeding activities that exploit the potential of crop wild relatives (CWR), and discuss how these are successfully faced once the unit of plant phenotyping becomes the individual plant grown as described. Results from our long-term research focusing on the application and further development of the principles related to the HSD and the prognostic breeding paradigm (Fasoula, 2013) in various crops and trials (Fasoula, 1990; Fasoula, 2004; Omirou et al., 2019) are summarized and placed in context.

## Barrier 1: Limited Seed Supply in the Segregating Generations Following a Cross

**Figure 1:**
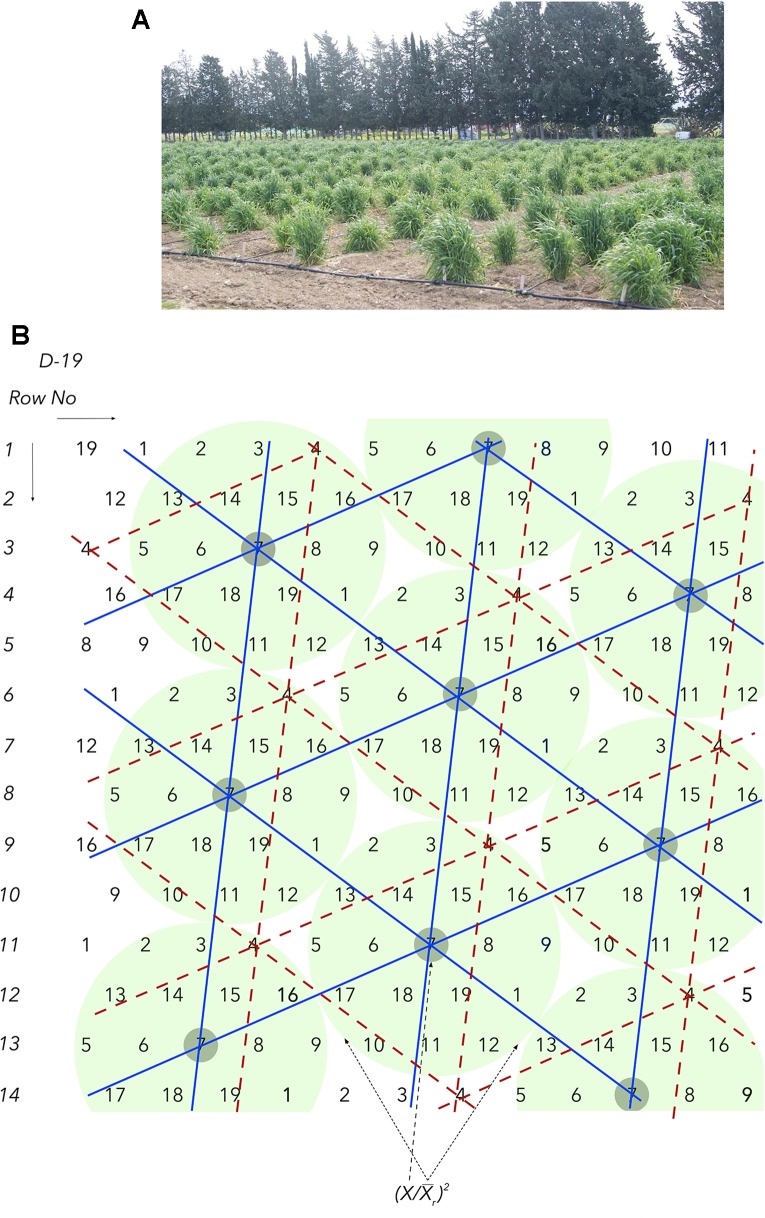
**(A)** All barley biomass and the multiple, fertile tillers (>70) in each field spot correspond to a single seed. The number of replications are commonly 40–120 and plant spacing is 100 cm. **(B)** The honeycomb selection design *D19* is used to demonstrate the principles of moving complete replicates and grids and the components of the prognostic equations.

This barrier relates to the limited seed supply in the early segregating generations following a cross and the fact that each seed represents a unique genotype. This obstacle, complicated by the next barriers, is so serious that it has led to the old, convenient, inexpensive, and common practice of visual selection (hence the renowned “breeder’s eye”) until enough homogeneous seed is gradually generated during the next few years, so as to permit replications, ranging between two and six, of densely grown plots. Additional testing locations are possible to be included only in the latter stages of the program. A similar problem of limited seed supply is encountered when crop wild relatives and *ex situ* materials in gene banks need to be phenotyped. Seed supplies are not an issue when working with individual plants in HSDs (**Figure 1**) and multi-location evaluations can successfully start as early as in F_2_ with a virtually unrestricted number of replications.

## Barrier 2: Effects of Interplant Competition

This barrier refers to the masking effects of interplant competition on selection efficiency (Kyriakou and Fasoulas, 1985; Fasoula, 1990; Fasoula and Fasoula, 1997a) and the dominating confusion regarding the concepts of competition and density that are commonly treated as one and the same. They are related, but not equivalent. This confusion further complicates the issue of associating the superiority ranking based on individual plant performance with the ranking under commercial stands. Competition is defined as “the plant-to-plant interference with the equal sharing of the density-limited growth resources caused by genetic and acquired differences and quantified by the CV of single-plant yields” (Fasoula and Fasoula, 1997a; Fasoula and Fasoula, 2002). It is possible and compatible to have high planting densities with simultaneous reduction of inter-plant competition, whereas performance under different densities, contrary to an existing belief, does not need to be represented in the analysis of crop yield potential (CYP).

Barrier 2 is overcome through a) the partitioning of CYP into components measured concurrently and precisely at the single-plant level under conditions excluding interplant competition (Fasoula and Fasoula, 2000; Fasoula and Fasoula, 2003) and b) the development of the whole-plant and sibling line field prognostic or phenotyping equations (Fasoula, 2006; Fasoula, 2008; Fasoula, 2013). The CYP components are condensed in two, the plant yield potential *per se* and the plant stability index, the latter being quantified on a single-plant basis for the first time. The whole-plant prognostic equation PPE incorporates the two components:

$$PPE={\left(x/{\bar{x}}_{r}\right)}^{2}{\left({\bar{x}}_{g}/s\right)}^{2}$$

where x is the plant yield in grams, ${\bar{x}}_{r}$ the mean yield in grams of the surrounding moving and complete circular replicate, ${\bar{x}}_{g}$ is the mean yield in grams of the sibling line plants allocated in a moving grid, and s its standard deviation. Thus, the need to identify an optimal planting density becomes ultimately unnecessary and the ranking under ultra-wide distances corresponds very well to the ranking under commercial densities (Fasoula and Fasoula, 2000; Fasoula, 2013; Greveniotis and Fasoula, 2016; Omirou et al., 2019).

We advocate that the critical question is not whether the entry ranking in densely grown plots corresponds to the ranking of individual plants in wide distances, but whether we truly need genotypes that behave differently under the two conditions. New varieties should have the genetic makeup that renders them density-neutral or density-independent (Fasoula and Fasoula, 2000; Fasoula and Fasoula, 2002; Fasoula, 2013; Fasoula et al., 2014; Uphoff et al., 2015). This is particularly necessary considering the abrupt fluctuations under climate change and for the drought-prone and marginal regions. Further and importantly, the dense planting in itself is simply neither conducive nor practical towards implementing field phenotyping of individual plant canopies and the corresponding entangled root systems for root phenotyping. An additional disturbing effect of interplant competition that hinders efficiency of selection relates to the existing negative correlation between yielding and competitive ability (Fasoula and Fasoula, 1997a and references therein).

## Barrier 3: Effects of Soil and Spatial Heterogeneity

This barrier relates to the masking effects of soil and spatial heterogeneity. The development of HSDs enables the effective sampling of soil heterogeneity, ensuring that all plants and sibling lines are allocated under comparable growing conditions in both fertile and non-fertile spots. In all HSDs, each plant in the trial is found in the middle of a i) circular, ii) complete, and iii) moving replicate and each sibling line belongs to a moving and triangular grid that covers uniquely the whole spectrum of spatial heterogeneity (**Figure 1A**). These properties come along with an unrestricted number of replications, commonly between 40 and 120, and the two precision field phenotyping equations.

## Barrier 4: Statistical Analysis Issues

This barrier concerns the incremental changes in the breeding process that are real but impossible to be detected and captured by traditional selection designs and statistical analysis that focus on statistical significance and analysis of variance. The analysis pertinent to the prognostic breeding paradigm is very sensitive about minor differences and the ranking is based on the unique plant and sibling line prognostic equation values during all stages of the breeding program (Fasoula et al., 2019). This fact both anticipates and converges with the most recent awareness and recommendations about the pitfalls of the notion of statistical significance. The notable book by Ziliak and McCloskey (2008) and the subsequent special issue of The American Statistician about the pitfalls and misuse of statistical significance (Wasserstein et al., 2019) are relevant. As the latter authors point out: “In sum, “statistically significant”—don’t say it and don’t use it”.

In prognostic breeding, the evaluated entries are recognized from the start as being inherently different, regardless of their potentially similar background. Each plant in the trial receives a unique quantitative identifier, its plant prognostic equation value, according to the exclusive properties of the HSDs. The ensuing ranking is based on accurate and quantitative evaluation standards that exclude any visual selection and breeder’s bias during all stages of the breeding program. Thus, it is the environment that decides about the “best” genotypes, not a subjective human preference.

Further details in Fasoulas and Fasoula (1995) describe the fundamental incompatibility between the analysis of variance (ANOVA) and the principles underlying the development of the HSDs. A small extract: “Experimental error, as estimated by variance analysis, is a pooled error based on the assumption that variances among entries are equal… however, entry variances are not equal, due to genetic differences that always exist among entries…instead of searching for procedures that reduce error variance by correcting the effects of spatial heterogeneity, plant breeders need procedures that exploit spatial heterogeneity to select for stability of performance early in the breeding program.”

The above have also implications towards novel approaches to explore the nature of gene action underlying quantitative traits (Fasoula and Fasoula, 1997b; Fasoula and Fasoula, 2002; Fasoula and Boerma, 2005). Of relevant interest also is recent work by Huang and Mackay (2016).

## Barrier 5: Differences Between Plants and Animals Affect Estimations of Genetic Gain and Response to Selection

Barrier 5 relates to the conditions that satisfy the so-called breeder’s equation, originally derived from the practice of animal breeding (Hill, 2014), that describes the expected response to selection and represents the presently established way to estimate genetic gain also in plant breeding. A simplified form of the equation (Falconer, 1989) is *R* = *σ*_p_ *h* ^2^ *i*, where *σ*_p_ is the population phenotypic standard deviation, *h* ^2^ the coefficient of heritability, and *i* the standardized selection differential. Briefly, the equation predicts that the larger the phenotypic standard deviation of the population under selection and the smaller the proportion of the (truly superior with greater number of progenies) plants advanced to the next generation, the higher will be the response *R* to selection. The same is expected when decreasing the generation time interval that is also related to the greater number of progenies.

In the practice of animal breeding, the unit of evaluation for selection has always been the individual animal raised with no competitive interactions for resources (Fasoulas and Fasoula, 1995), as each animal receives an own and specified feed portion. Falconer (1989) explicitly refers to this difference when discussing the equation. It follows that the most relevant conditions to apply the equation in plant breeding is when the individual plant is grown in conditions that exclude competitive interactions (**Figure 1A**). Even so, this form of the equation does not exploit the unique differences between plants and animals, such as the stationary nature of plants and the fact that the average plant produces a far greater number of progenies than an animal. The plant prognostic equation can be used effectively to select for high crop yield potential and, therefore, high response to selection, as discussed in details in Fasoula (2013). The derivation of PPE satisfies the conditions of the currently used “breeder’s equation” and at the same time explicitly takes into consideration the unique features of plants. Thus, it becomes feasible to report major gains (Fasoula, 2012; Greveniotis and Fasoula, 2016; Omirou et al., 2019) and overcome the current stagnation of yield gains of major crops that is widely recognized at around 1% (Peng et al., 2000; CGIAR, 2016). Further and also related to Barrier 6, there are substantial differences between the reproductive lineages of plants and animals. We assert that these differences have consequences for plant breeding, as the favorable events during plant development need to be captured at the individual plant level.

## Barrier 6: Plant (EPI)GENOMICS Is Based on Individual Genomes, But Not Plant Field Phenomics

This important barrier (Fasoula and Fasoula, 2000) concerns the fact that while all (epi)genomic analysis concern individual genomes, the corresponding current practices in plant breeding aiming to bridge the so-called genotype–phenotype gap concern multi-plant grown plots. Relevant articles that confirmed this concept and identified genomic variation among individuals of a variety include Haun et al. (2011) and Yates et al. (2012).

## Barrier 7: Automation Challenges

This barrier relates to the reasons underlying the lack of automation in plant breeding coupled with the lack of standards in the phenotyping trials. The adoption of automated phenotyping in plant breeding is still in its infancy (Walter et al., 2017). The need for standardization requirements is commonly expressed by members of the phenotyping community, although it is aptly recognized that “standardized methods are valuable, but novel methods are, too!…” (Lawrence-Dill et al., 2018). The application of the highly systematic HSD and the prognostic equations for unbiased selection of superior plants carry the intrinsic capability towards completely automizing the genetic improvement of crops from the earliest to the latest stages of a breeding program. This has immediate and highly positive impact towards the maximization of selection efficiency and the cost reduction of the produced seed. The allocation of individual plants in each HSD is highly regular, symmetrical, with repeating motifs, and independent of the trial location, thus overcoming the need for separate entry randomization in each location. Plants are allocated in horizontal rows in an ascending numerical order, permitting the creation of moving complete replicates across all levels of spatial heterogeneity and in all types of environments form marginal to highly productive.

Each plant in a HSD trial possesses a unique position identification number, for example 8-12-7, where number 8 gives the number of the horizontal row, number 12 gives the plant position on the row, and number 7 gives the number of the design code corresponding to the particular sibling line. Attached to this number is the corresponding unique value of the plant phenotyping or prognostic equation. The intrinsic properties of the designs that provide a matrix of standardized motifs across environments and the wide distances between plants facilitate the use of geo-referencing methods. Plants are ranked according to the value of their phenotyping equation and selection of the “best” in each environment is an unbiased process, rendering the same results regardless of the person performing the analysis. It is thus amenable to full automation and robotization.

Following the discussion on the barriers, we would also like to draw some attention to some recent references and work that support the above and highlight the novel possibilities that unfold. There is a recent acknowledgment that “it has become measurably harder to generate ideas and new approaches that result in real gains” (Lawrence-Dill et al., 2019) and the endorsement by Fischer et al. (2019) of the “unique breeding strategy proposed in the 1970s by Professor AC Fasoulas …”. Although the latter article appears to ignore all literature and critical developments in the strategy after 1993, at the risk of diverting resources towards the already known, it offers an interesting realization by experienced plant physiologists/breeders. Further, different groups have validly acknowledged that the physiology-based breeding that relies on measurements of secondary traits presumed to be proxies for crop yield has been largely unsuccessful (NSF, 2011), while in the prognostic breeding applications, the direct and successful selection for yield is the norm.

Importantly, there is good convergence of the concepts described above and the highly successful agronomic practices of the System of Rice Intensification (SRI) (Uphoff et al., 2015). Thus, the above ideas can be applied towards additional benefits for improving the agronomic performance of crops and contributing to the minimization of the global yield gaps.

## Conclusions

The field phenotyping community can benefit by giving due consideration to the suggested innovations towards overcoming current barriers in plant phenotyping and phenomics, while serving also the developments in plant (epi)genomics. The involvement of international, multidisciplinary teams will contribute to the deeper understanding of the methodology. This will, in turn, unfold additional options and facilitate the successful automation, standardization, and robotization of large-scale phenotyping for plant breeding.

## Author Contributions

DF and MO conceived the work and all authors contributed to the final form of the manuscript and securing relevant funding.

## Funding

The Cyprus Research Promotion Foundation in the frame of the research project MAGNET (INFRASTRUCTURES/1216/0032) supported this work.

## Conflict of Interest

The authors declare that the opinion article was written in the absence of any commercial or financial relationships that could be construed as a potential conflict of interest.
